# Overview of Explainable Artificial Intelligence for Prognostic and Health Management of Industrial Assets Based on Preferred Reporting Items for Systematic Reviews and Meta-Analyses

**DOI:** 10.3390/s21238020

**Published:** 2021-12-01

**Authors:** Ahmad Kamal Mohd Nor, Srinivasa Rao Pedapati, Masdi Muhammad, Víctor Leiva

**Affiliations:** 1 Mechanical Department, Universiti Teknologi Petronas, Seri Iskandar 32610, Malaysia; srinivasa.pedapati@utp.edu.my (S.R.P.); masdimuhammad@utp.edu.my (M.M.); 2 School of Industrial Engineering, Pontificia Universidad Católica de Valparaíso, Valparaíso 2362807, Chile; victor.leiva@pucv.cl

**Keywords:** AI, explainable deep learning, machine learning, PHM, PRISMA, reliability, sensing and data extraction, XAI

## Abstract

Surveys on explainable artificial intelligence (XAI) are related to biology, clinical trials, fintech management, medicine, neurorobotics, and psychology, among others. Prognostics and health management (PHM) is the discipline that links the studies of failure mechanisms to system lifecycle management. There is a need, which is still absent, to produce an analytical compilation of PHM-XAI works. In this paper, we use preferred reporting items for systematic reviews and meta-analyses (PRISMA) to present a state of the art on XAI applied to PHM of industrial assets. This work provides an overview of the trend of XAI in PHM and answers the question of accuracy versus explainability, considering the extent of human involvement, explanation assessment, and uncertainty quantification in this topic. Research articles associated with the subject, since 2015 to 2021, were selected from five databases following the PRISMA methodology, several of them related to sensors. The data were extracted from selected articles and examined obtaining diverse findings that were synthesized as follows. First, while the discipline is still young, the analysis indicates a growing acceptance of XAI in PHM. Second, XAI offers dual advantages, where it is assimilated as a tool to execute PHM tasks and explain diagnostic and anomaly detection activities, implying a real need for XAI in PHM. Third, the review shows that PHM-XAI papers provide interesting results, suggesting that the PHM performance is unaffected by the XAI. Fourth, human role, evaluation metrics, and uncertainty management are areas requiring further attention by the PHM community. Adequate assessment metrics to cater to PHM needs are requested. Finally, most case studies featured in the considered articles are based on real industrial data, and some of them are related to sensors, showing that the available PHM-XAI blends solve real-world challenges, increasing the confidence in the artificial intelligence models’ adoption in the industry.

## 1. Introduction

### 1.1. General Progress in Artificial Intelligence

Artificial intelligence (AI) continues its extensive penetration into emerging markets, driven by untapped opportunities of the 21st century and backed by steady and sizeable investments. In the last few years, AI-based research shows much concentration in areas such as large-scale machine learning (ML), deep learning (DL), reinforcement learning, robotic, computer vision, natural language processing, and internet of thing [1]. 

According to the first AI experts report in the “One-hundred-year study on artificial intelligence”, AI ability will be heavily embodied in education, healthcare, home robotics, safety, security, and transportation, as well as entertainment, in North American cities by the 2030s [1].

The increasing data volume [2] and breakthrough in ML, coupled with the pressing need to be more efficient and innovatively democratize AI to the global scene, are currently relevant. A survey conducted by McKinsey [3] (www.mckinsey.com, accessed on 25 November 2021) recorded an annual increase of 30% in AI investment from 2010 to 2013 and 40% from 2013 to 2016. In 2016, the total global investment amounted from 26 to 39 billion dollars by tech firms and external investments. In 2030, AI could potentially be valued up to 15 trillion dollars in global gross domestic product growth thanks to automation and product innovation, while reducing approximately seven trillion dollars in operational costs [4]. AI-driven technology leads to an incremental change in labor market requirement, where increasing technological ability, together with higher cognitive and social-emotional skills, are needed to support AI-based infrastructures, whereas manual and basic cognitive skills experience less demand [5].

AI is a technical discipline defined as the science of making computers do things that would require intelligence if done by humans [6]. The reasoning of AI imitates natural laws translated into working algorithms [7]. Some important fields in AI research include expert systems, consisting of rule-based reasoning, case-based reasoning, and fuzzy systems, along with ML models [8,9,10], such as an artificial neural network (ANN), support vector machine, DL, and heuristic algorithms [11,12]. The availability of the parallel graphics processing unit and open-source development tools unlock the door for literally everyone to solve technical challenges, sometimes surpassing human performance [13,14]. These abilities and specialized tools make AI so appealing in technically infused domains such as computer vision [13], healthcare [6], image processing [7], and reliability engineering [11].

### 1.2. Artificial Intelligence in Prognostic and Health Management

ML, in general, and more specifically DL, are part of the reliability research landscape, including prognostic and health management (PHM) [15,16,17]. PHM provides guidelines and frameworks to safeguard the healthy state of assets. PHM minimizes risks, maintenance costs, and workload, thus optimizing maintenance activities. PHM is defined by standards of the Institute of Electrical and Electronics Engineers (IEEE) as “a maintenance and asset management approach utilizing signals, measurements, models, and algorithms to detect, assess, and track degraded health, and to predict failure progression” [18]. Accordingly, three types of PHM activities are distinguished: (i) prognostic, (ii) diagnostic, and (iii) anomaly detection. Prognostic is the action of determining the remaining useful life (RUL) or the leftover operational time of an asset before a failure [17]. Diagnostic is the action of classifying a failure and, to some extent, discovering the detailed root cause of this failure [19]. Anomaly detection consists of identifying unusual patterns going against the normal behavior of operational indicators [20].

A considerable part of the literature supports the idea of AI as being at the forefront in PHM studies [15,20]. To mention a few: (i) long short-term memory (LSTM) ANN was employed in [21] with degradation image to estimate the RUL of rotating machinery; (ii) a regression tree was used to predict the RUL of central heating and cooling plant equipment in [22]; (iii) the combination of logistic regression with L2 SVM was proposed for gas circulator unit prognostic [23,24]; (iv) random forest was utilized to diagnose fault for semiconductor equipment failure in [25]; (v) convolutional and fully connected layers with Softmax activation were considered in [26] to diagnose rotating machine issues; and (vi) gradient-boosted decision trees outperformed other methods in the anomaly detection of hard drives in [27].

### 1.3. Black-Box Artificial Intelligence Problem

Though very powerful, many AI methods are black boxes in nature, meaning that the inner mechanism to produce outputs in these methods are unknown [28,29]. Obviously, this opacity is an obstacle in AI penetration across many sensitive or high-stake areas such as banking, defense, finance, and medical areas, even in the common industry [30,31]. The end-users and experts of the domain in question need the assurance that the model’s inner process is understandable [32]. Such an opaqueness adds operational and confidentiality hazards, bias, or nonethical outputs risks [33]. The lack of transparency discourages responsible exploitation of AI decisions [34], model troubleshooting [35], and improvement [32]. Moreover, it further complicates the question of responsibility ownership in the case of wrong decision [36]. Therefore, with the increasing scrutiny and regulation on AI usage, the need to make AI methods as transparent as possible is pressing. This includes the general data protection regulation in the European Union and the ethics guidelines for trustworthy AI presented by the European Commission High-Level Expert Group on AI [37,38,39].

### 1.4. The Need for Explainable Artificial Intelligence

Explainable artificial intelligence (XAI) is a discipline dedicated in making AI methods more transparent, explainable, and understandable to end-users, stakeholders, nonexperts, and non-stakeholders alike to nurture trust in AI. The growing curiosity in XAI is mirrored by the spike of interest in this search term since 2016 and the rising number of publications throughout the years [38].

The Defense Advanced Research Projects Agency (DARPA) developed the XAI Program in 2017, while the Chinese government announced the Development Plan for New Generation of Artificial Intelligence in the same year, both promoting the dissemination of XAI [40]. The general needs for XAI are as follows:

(i) Justification of the model’s decision by identifying issues and enhancing AI models.
(ii) Obedience of the AI regulation and guidelines in usage, bias, ethics, dependability, accountability, safety, and security.
(iii) Permission for users to confirm the model’s desirable features, promote engagement, obtain fresh insights into the model or data, and augment human intuition.
(iv) Allowance for users to better optimize and focus their activities, efforts, and resources.
(v) Support for the model development when it is not yet considered as reliable.
(vi) Encouragement for the cooperation between AI experts and external parties.

### 1.5. Common XAI Approaches

While there are many definitions linked to XAI, this work concentrates only on the most employed notions of interpretability and explainability. On the one hand, interpretability refers to the ability to provide human-understandable justification for the one’s behavior. Thus, interpretable AI points to the model’s structures which are transparent and readily interpretable. On the other hand, explainability describes an external proxy used to describe the behavior of the model. Hence, explainable AI refers to post-hoc approaches utilized for explaining a black-box model. The first definition explicitly distinguishes between black-box and interpretable models. The second definition takes a broader connotation where explainability is accented as a technical ability to describe any AI model in general and not only black-box identification.

XAI approaches are classified according to an explanation scope [41]. Intrinsic models are interpretable due to their simplicity such as in linear regression and logic analysis of data (LAD), while post-hoc approaches interpret more complex nonlinear models [32,33]. Examples of post-hoc approaches are local interpretable model-agnostic explanations (LIME) and Shapley additive explanations (SHAP).

An approach can be categorized as (i) AI-model specific or (ii) employable in any AI model or model agnostic [14,42]. Class activation mapping (CAM), for example, can only be utilized after CNN. Layer-wise relevance propagation (LRP) and gradient-weighted CAM may be employed in any gradient-based models.

Therefore, the explanation by the XAI model can either cater to local data instances or to the whole (global) dataset [41]. For example, SHAP may generate both local and global explanations, while LIME is only suitable for local explanation.

### 1.6. Review Motivation

The main objective of this work is to present an overview of XAI applications in PHM of industrial assets by using preferred reporting items for systematic reviews and meta-analyses (PRISMA, available online: www.prisma-statement.org, accessed on 4 October 2021) guidelines [43]. PRISMA is an evidence-based guideline that ensures comprehensiveness, reducing bias, increasing reliability, transparency, and clarity of the review with minimum items [44,45]. PRISMA is a 27-checklist guideline that needs to be satisfied as best as possible for the best practice in systematic review redaction. However, in the systematic review presented in the present study, items 12, 13e, 13f, 14, 15, 18–22, and 24 of the PRISMA methodology were omitted as they were not dealt with here; see prisma-statement.org/PRISMAstatement/checklist.aspx (accessed on 19 November 2021) for details on these items.

**Figure 1:**
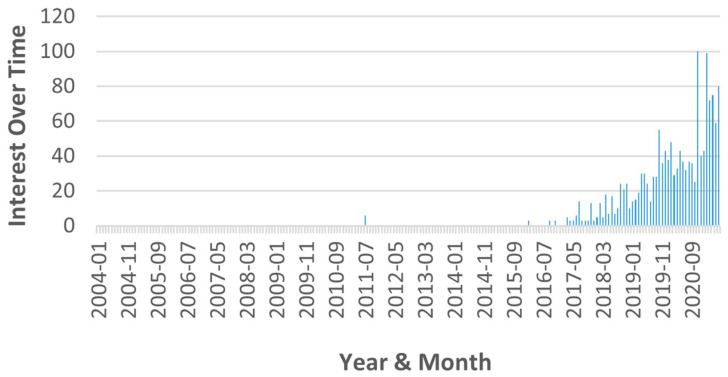
Interest shown for the term “explainable AI” in Google searches.

The rationalities motivating the compilation of this review are the following:

(i) Global interest in XAI: According to our survey, the general curiosity toward XAI has surged since 2016 [14]. Figure 1 shows the interest expressed for the term “explainable AI” in Google searches, with 100 being the peak popularity for any term.
(ii) Specialized reviews: In the early years, several general surveys on XAI methods were written [32,34]. More recently, as the discipline grows, more specialized works emerged. Reviews on XAI have been related to drug discovery [31], fintech management [35], healthcare [30,33,36], neurorobotics [39], pathology [28], plant biology [37], and psychology [29]. Thus, it is necessary to produce an analytical compilation of PHM-XAI works, which is still absent.
(iii) PHM nature and regulation: PHM is naturally related to high-investment and safety-sensitive industrial domains. Moreover, it is pressing to ensure the use of well-regulated AI in PHM. Hence, it is necessary for XAI to be promoted as much as possible and its know-how disseminated for the benefit of PHM actors.

The review goals are achieved by addressing the following points:

(i) General trend: This is related to an overview of the XAI approach employed, the repartition of the mentioned methods according to PHM activities, and the type of case study involved.
(ii) Accuracy versus explainability power: According to DARPA, the model’s accuracy performance is inverse to its explainability prowess [40].
(iii) XAI role: This must assist or overload PHM tasks.
(iv) Challenges in PHM-XAI progress: Crosschecks were done with the general challenges raised in [14,32,34,38] associated with:
  (a) The lack of explanation evaluation metrics.
  (b) The absence of human involvement for enhancing the explanation effectivity.
  (c) The omission of uncertainty management in the studied literature.

The remainder of this paper is organized as follows: In Section 2, the methodology is introduced, followed by the results presentation in Section 3. Then, the discussion is elaborated in Section 4. Finally, the concluding remarks are presented in Section 5.

## 2. Methodology

### 2.1. Framework

A single person performed the search, screening, and data extraction of the articles considered in this study. Thus, no disagreement occurred in all the steps mentioned. Only peer-reviewed journal articles on PHM-XAI of industrial assets between 2015 and 2021 in English language were selected.

### 2.2. Databases

Five publication databases consisting of ScienceDirect of Elsevier (until 17 February 2021), IEEE Xplore (until 18 February 2021), SpringerLink (until 22 February 2021), Scopus (until 27 February 2021), and Association for Computing Machinery (ACM) Digital Library (until 28 May 2021) were explored. Advanced search was used, but since the database features are different, a specific strategy was adopted. In IEEE Xplore, search was conducted in the “abstract” and “document title” fields only as they are the most relevant options. The database also authorizes search within the obtained results in the “search within results” field. Wildcard was not used in IEEE Xplore even though it was permitted. Comprehensive search in the “title”, “abstract”, and “keywords” fields were performed in ScienceDirect and Scopus; “title”, “abstract”, and “author-specified keywords” fields for ScienceDirect; and “search within article title”, “abstract”, and “keywords” fields for Scopus. However, unlike Scopus, ScienceDirect does not support wildcard search; therefore, it was only employed in Scopus. In SpringerLink, the “with all the words” field was utilized altogether with wildcards. In ACM, both the ACM full-text collection and ACM guide for obtaining the literature were examined. The “Search within” option in the “title”, “abstract”, and “keywords” was executed with wildcard. Once performed, the screening of duplications was performed by using the Zotero software (www.zotero.org, accessed on 4 October 2021). The full research strategy is listed in Appendix A.

### 2.3. Steps of Our Bibliographical Review

The following screening steps were executed one after another for obtaining a result, with each screening step starting in the title, then the abstract, and next the keywords:

(S1) Verify whether the article type is research or not.
(S2) Exclude non-PHM articles by identifying absence of commonly employed PHM terms such as prognostic, prognosis, RUL, diagnostic, diagnosis, anomaly detection, failure, fault, or degradation.
(S3) Discard non-XAI articles by identifying absence of commonly used XAI terms which are explainable, interpretable, and AI.
(S4) Eliminate non-PHM-XAI articles by identifying the absence of both PHM and XAI terms as, respectively, indicated in steps (ii) and (iii) above.
(S5) Remove articles related to medical applications or network security.

Then, the context of the articles was examined on the remaining works for final screening and so to retain only the desired articles. The data extracted from the articles were gathered in a Microsoft Excel file with each column corresponding to each investigated variable. Directly retained variables were: “author”, “publication year”, “title”, “publisher”, and “publication/journal name”. Further information extracted from the article context analysis is as follows:

(i) PHM activity category: This corresponds to either anomaly detection, prognostic, or diagnostic, with structural damage detection as well as binary failure prediction being considered as diagnostic.
(ii) XAI approach employed: This is related to the category of the XAI method.
(iii) Recorded performance: This is associated with the reported result. Some papers clearly claim the comparability or the superiority of the proposed method over other tested methods. In the case where comparison was not conducted, the reported standalone results for accuracy, precision, F1 score, area under the receiving operating characteristic curve (AUC) score, area under precision-recall curve (PRAUC) score, or the Cohen kappa statistic score were referred to Table A4 in Appendix A and classified as either “bad”, “fair”, “good”, and “very good”. When mixed performance of good and very good was recorded for the same method, it was quantified as only “good”. When a method was superior to the rest, it was classified as “very good” unless detailed as only “good”. Some results were appreciated based on the problem at hand, for example using the mean square error (MSE), root mean square error (RMSE), and mean absolute error (MAE) as direct comparisons is not possible.
(iv) XAI role in assisting PHM task: This regards the role of XAI in strengthening PHM ability.
(v) Existence of explanation evaluation metrics: This is stated as presence or not of a metric.
(vi) Human role in PHM-XAI works: This is considered as existence of the mentioned role or not.
(vii) Uncertainty management: This is linked to if uncertainty management in any of the stages of the PHM or XAI approaches increases the possibility for adoption by user due to additional surety.
(viii) Case study type (real or simulated): Real was considered when the data of a case study came from a real mechanical device, whereas simulated was considered when data were generated utilizing any type of computational simulation.

### 2.4. Outputs

The outputs were presented in the following forms:

(i) Table: Selected and excluded articles with variables sought.
(ii) Pie chart: Summary of the PHM activity category, explanation metric, human role, and uncertainty management.
(iii) Column graph: Summary of the PHM-XAI yearly trend, XAI approach employed, recorded performance, and XAI role in assisting a PHM task.

## 3. Results

### 3.1. Framework

We selected 3048 papers from the databases according to the applied keywords with their respective number (absolute frequency) as shown in Table A3 of Appendix A. Note that 288 articles were screened out as duplicates. Out of the 2760 remaining, 25 papers were screened out as they are editorial papers or documents related to news. Then, 70 papers were selected according to criteria (S1)–(S5) described in Section 2.3 (steps of our bibliographical review) from the remaining 2735 articles. Lastly, only 35 papers were selected as other 35 articles were deemed not relevant with the reviewed topic after context verification. The final selected and excluded studies can be found, respectively, in Table A1 and Table A2 of Appendix A.

### 3.2. PRISMA Flow Diagram

**Figure 2:**
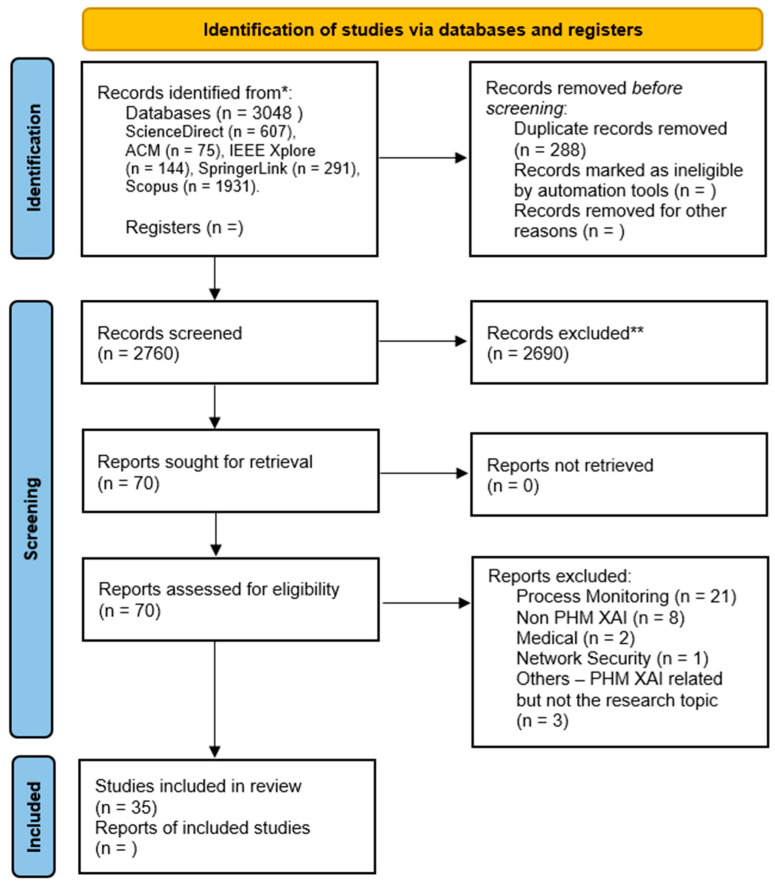
PRISMA flow diagram of the search strategy for our review on PHM-XAI.3.3 (“*” indicates that “n = ” in the database field corresponds to the total number of records from all the databases specified below; and “**” states that the Zotero software was used for duplication analysis).

As mentioned, the selected and excluded articles based on the criteria for inclusion are disclosed, respectively, in Table A1 and Table A2. The PRISMA flow diagram of the selection and screening processes is displayed in Figure 2.

**Figure 3:**
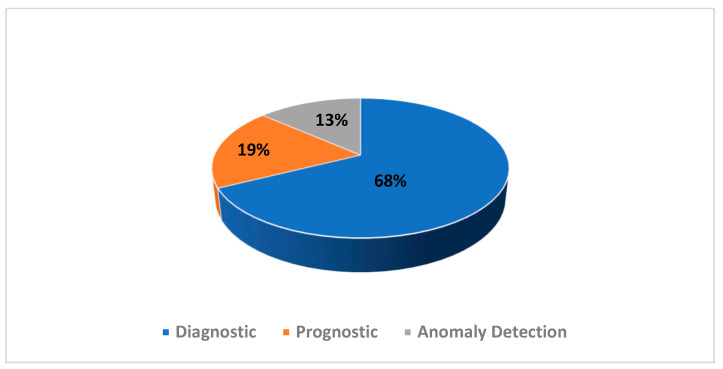
Distribution of PHM tasks for the selected articles.

**Figure 4:**
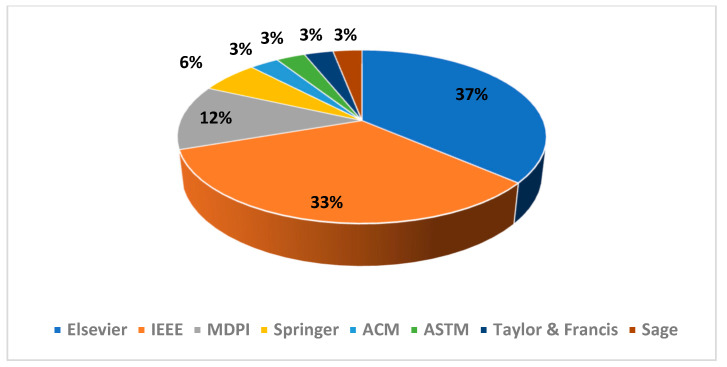
Distribution of the selected articles according to the indicated publisher.

**Figure 5:**
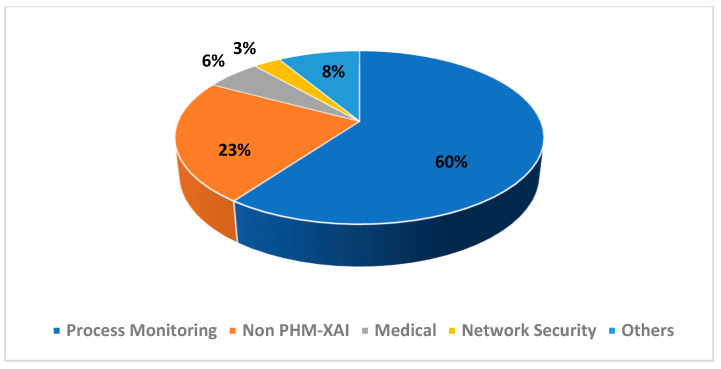
Distribution of the excluded articles according to the topic.

**Figure 6:**
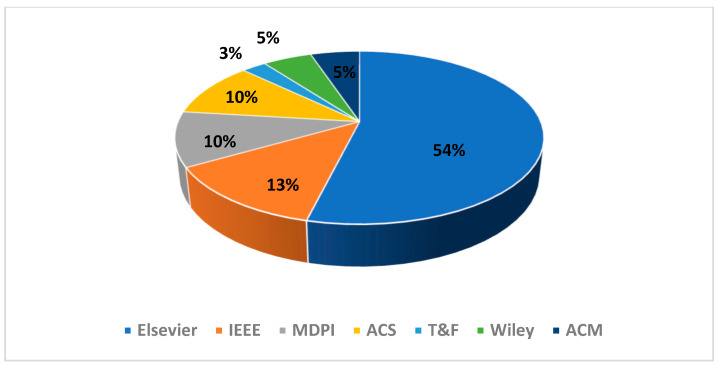
Distribution of the excluded articles according to the publisher.

The repartition of the selected articles’ PHM domain as well as their publisher are presented in Figure 3 and Figure 4, respectively. The repartition of the excluded articles’ PHM domain as well as their publisher are presented in Figure 5 and Figure 6, respectively. As noted from Figure 3, diagnostic research holds the biggest share in PHM-XAI articles. Figure 4 illustrates IEEE and Elsevier publishers as being the biggest sources of the accepted articles.

Numerous unselected publications, though related to XAI, correspond to process monitoring research, as shown in Figure 5. These works were excluded as they are closely related to quality context rather than failure of products. Some works are focused on products instead of the industrial assets. Furthermore, the anomaly described is seldom associated with process disturbance rather than failure degradation. Studies concerning the network security were also omitted. In addition, most of the excluded articles come from the Elsevier and IEEE publishers as confirmed by Figure 6, further showing that these publishers are the main sources of many XAI-related articles.

## 4. Discussion

### 4.1. General Trend

**Figure 7:**
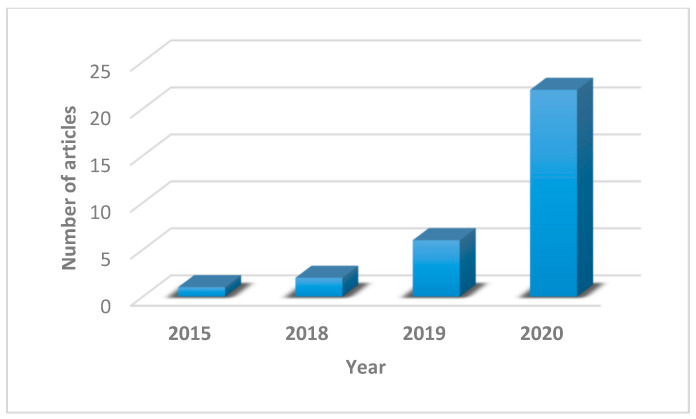
Distribution of the selected articles according to the indicated year.

As shown in Table A1 of Appendix A and summarized in Figure 7, the accepted articles according to the publication year show an upward trend, with a major spike in 2020, indicating a growing interest in XAI from the PHM researchers. However, the number of accepted articles is still very small, reflecting the infancy state of XAI in PHM, compared to other research fields such as cyber, defense, healthcare, and network securities. XAI is especially beneficial to the latter domains as it helps in fulfilling their primary functions of protecting lives and assets—contrasted to PHM research, where it is predominantly focused in facilitating financial decision making. In the healthcare field, for example, the efforts to evaluate explanation quality are presently an active topic, which is not the case of PHM [46]. The understanding of XAI is also limited in PHM, partly due to comprehensible distrust in using AI in the first place, compounded with the amount of investment needed to build AI systems that is yet to be proven in real life. In fact, manufacturing and energy sectors, associated closely with PHM, are amongst the slowest in adopting AI [47]. Thus, AI only thrives in PHM research. In brief, more exposure and advocation of XAI in PHM are needed to nurture trust in the AI usage, improving day to day the operational efficiency and enabling the overall safeguard of industrial assets and lives.

Note that 70% of the included PHM-XAI works come from ScienceDirect and IEEE Xplore as testified by Figure 4. Most of the excluded articles in the final stage also come from the mentioned databases as shown in Figure 6. These observations suggest that these two databases concentrate XAI-related works. It is commendable for a specialized journal in other publishers to promote the use of XAI in PHM through dedicated symposiums and special issues, which are still scarce.

### 4.2. XAI

**Figure 8:**
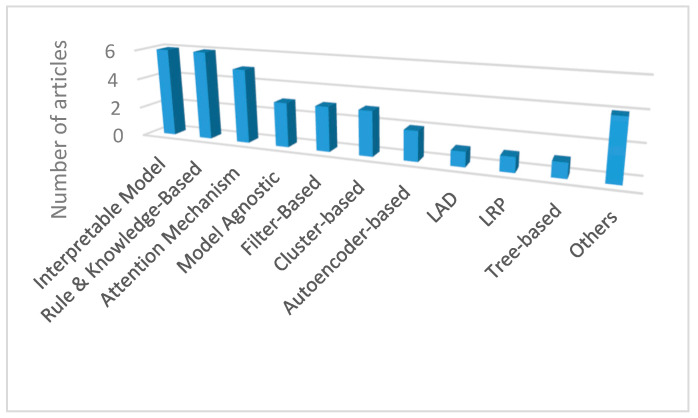
Distribution of the XAI approach type in the selected articles.

Interpretable models, rule- and knowledge-based models, and the attention mechanism are the most employed methods as illustrated in Figure 8. These methods existed well before XAI become mainstream. Then, their implementations became well documented and common. Interpretable approaches consist of linear models widely used before the introduction of nonlinear models. Rule- and knowledge-based models possess the traits of expert systems which became widespread earlier and led to the popularity of AI [48]. The attention mechanism was developed in the image recognition field to improve classification accuracy [49].

Other techniques such as model agnostic explainability and LRP are less explored but are anticipated to permeate in the future due to their nature. They could be used with any black-box models. Furthermore, the performance of the AI models is not altered by these techniques. Model agnostic acts as an external method to the model to be explained while LRP requires only the gradient flow of the network. LAD is another interesting technique due to its potential combination with fault tree analysis that is seldom utilized in complex risk management such as in the aerospace and nuclear industries. The lack of coverage in LAD entails more investigation from the researchers on this topic.

**Figure 9:**
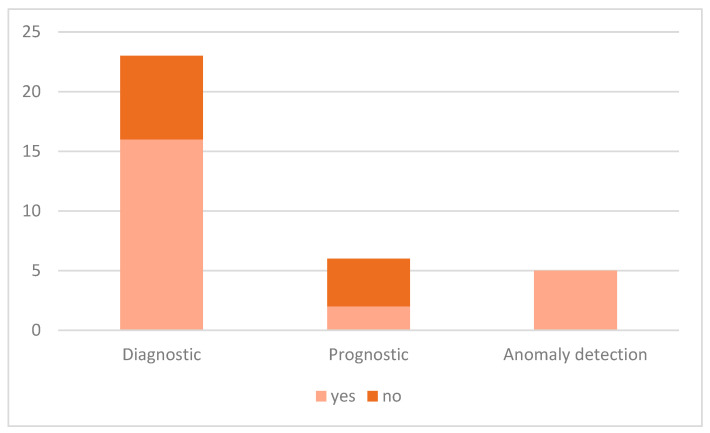
Distribution of the XAI assistance in the indicated PHM task.

The diagnostic domain occupies the majority share amongst the accepted works as presented in Figure 3. Looking at the XAI-assisted PHM column in Table A1 of Appendix A, it can be deduced that XAI boosts diagnostic ability. Drawing a parallel between the information from Figure 3 and Table A1, it may be inferred that XAI is particularly appealing to diagnostic as it can be applied directly as a diagnostic tool or in addition to other methods. XAI could provide additional incentive to diagnostic whose main objective is to discover the features responsible for the failure as shown in Figure 9. This interesting point signifies that the diagnostic tasks in these papers are dependent on XAI. Therefore, XAI is not only a supplementary feature in diagnostics but also an indispensable tool. The same phenomenon is observed in anomaly detection as presented in Figure 9. Knowing the cause of anomaly could potentially avoid false alarms, preventing resource wastage. Thus, XAI might be employed to execute PHM tasks and explain them.

**Figure 10:**
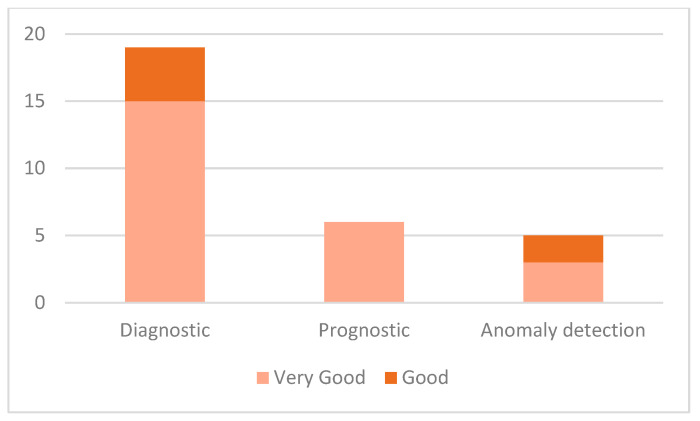
Distribution of the performance of AI models according to the indicated task.

Table A1 reveals that some XAI approaches directly assist the PHM tasks achieving excellent performance. Furthermore, the recorded PHM performance of both XAI and non-XAI methods (works that depend on XAI for explanation only) are mostly very good for diagnostics and prognostics, as depicted in Figure 10. In brief, no bad results were recorded as confirmed by Figure 10. Whether the results are contributed by XAI or not, it can safely be concluded that explainability does not affect the tasks’ accuracy in the studied works. The outcomes and reported advantage of XAI as a PHM tool are important steps in eradicating the skepticism and mistrust of the industry in the AI usage. These facts might intensify the assimilation of AI in the industry.

### 4.3. PHM

**Figure 11:**
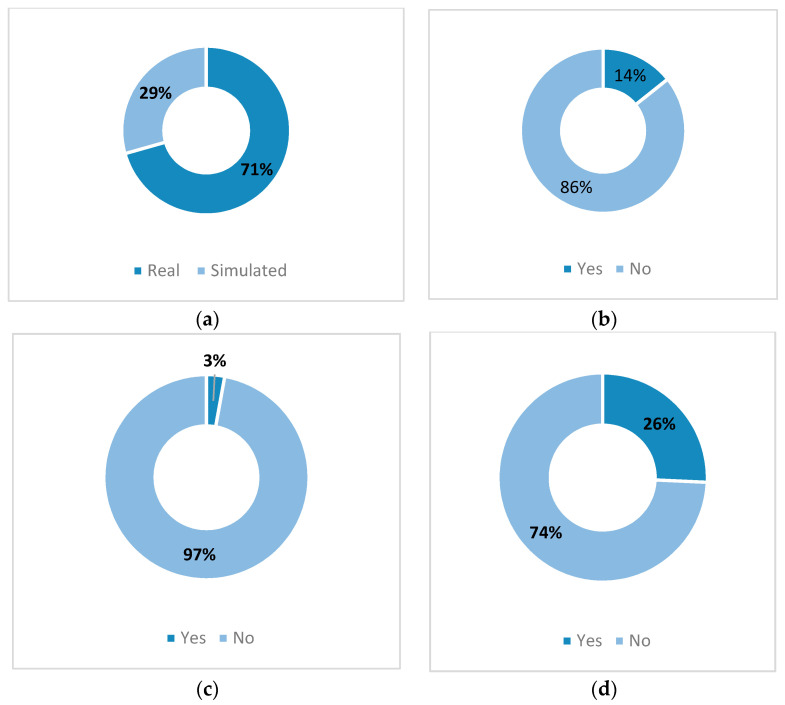
(**a**) Distribution of the type of case study in the selected articles; (**b**) distribution of human involvement (yes/no) in the selected articles; (**c**) distribution of explanation metric inclusion (yes/no) in the selected articles; and (**d**) distribution of uncertainty management inclusion (yes/no) in the selected articles.

Real industrial data are mostly used in case studies to demonstrate the effectiveness of XAI as reflected in Figure 11a. Furthermore, the studies reflect the outreach of XAI in diverse technical sectors such as aerospace, automotive, energy, manufacturing, production, and structural engineering fields. These positive outlooks prove that the available PHM-XAI combinations are suitable to solve real-world industrial challenges with at least a good performance, boosting the confidence in the AI models’ adoption.

### 4.4. Lack of Current Studies

#### 4.4.1. Human Role in XAI

A very small role was played by humans in the examined works as illustrated in Figure 11b. Human participation is vital for evaluating the generated explanation, as it is intended to be understood by them. This involvement helps in the assimilation of other human-related sciences to PHM-XAI such as human reliability engineering (HRA), psychology, or even healthcare, further enriching this new field [50]. Furthermore, human involvement is encouraged for the development of interactive AI, where the expert’s opinion strengthens or debates the generated explanation, presenting an additional guarantee in AI performance.

#### 4.4.2. Explainability Metrics

Note that the usage of explanation evaluation metrics is nearly nonexistent as presented in Figure 11c. The explanation evaluation method engineered for the PHM usage is practically absent according to our study. These metrics are vital to the researchers and developers when evaluating the explanation quality. It is recommended that adequate assessment metrics for PHM explanation, considering security and safety risk, maintenance cost, time, and gain are developed and adopted. Such metrics should require the collaboration of all PHM actors to satisfy the need of each level of hierarchy. From this angle, XAI experts could be inspired by the work performed in the HRA domain, which studies the human-machine interaction in reliability perspective [50]. An overview of explanation metrics and methods is presented in [51], whereas the effectiveness of explanation from experts to nonexperts is studied in [52], and a metric to assess the quality of medical explanation was proposed in [53].

#### 4.4.3. Uncertainty Management

Various types of uncertainty management methods are adopted in different stages in the studied works on the PHM-XAI area as detailed in Table A1. Nevertheless, note that, in Figure 11d, much improvement is still required in this area. Uncertainty management gives additional surety to users to adopt PHM-XAI methods compared to point estimation models. Furthermore, uncertainty quantification is vital to provide additional security to AI infrastructure against adversarial examples, either unintentionally or motivated by the attack. This quantification might minimize the risk of wrong explanation being produced from unseen data due to adversarial examples.

## 5. Conclusions

In this work, a state-of-the-art systematic review on the applications of explainable artificial intelligence linked to prognostics and health management of industrial assets was compiled. The review followed the guidelines of preferred reporting items for systematic reviews and meta-analyses (PRISMA) for the best practice in systematic review reporting. After applying our criteria for inclusion to 3048 papers, we selected and examined 35 peer-reviewed articles, in the English language, from 2015 to 2021, about explainable artificial intelligence related to prognostics and health management, to accomplish the review objectives.

Several interesting findings were discovered in our investigation. Firstly, this review found that explainable artificial intelligence is attracting interest in the domain of prognostics and health management, with a spike in published works in 2020, though still in its infancy phase. The interpretable model, rule- and knowledge-based methods, and attention mechanism are the most widely used explainable artificial intelligence techniques applied in the works of prognostics and health management. Secondly, explainable artificial intelligence is central to prognostics and health management, assimilated as a tool to execute such tasks by most diagnostic and anomaly detection works, while simultaneously being an instrument of explanation. Thirdly, it was discovered that the performance of prognostics and health management is unaltered by explainable artificial intelligence. In fact, the majority of works that related both approaches achieved excellent performance while the rest produced only good results. However, there is much work to be conducted in terms of human participation, explanation metrics, and uncertainty management, which are nearly absent.

This overview discovered that most real, industrial case studies belonging to diverse technical sectors are tested to demonstrate the effectiveness of explainable artificial intelligence, signifying the outreach and readiness of general artificial intelligence and explainable artificial intelligence to solve real and complex industrial challenges.

The implications of this study are the following:

(i) PHM-XAI progress: Much unexplored opportunity is still available for prognostics and health management researchers to advance the assimilation of explainable artificial intelligence in prognostics and health management.
(ii) Interpretable models, rule- and knowledge-based models, and attention mechanism: These are the most widely used techniques and more research involving other approaches could give additional insight into the prognostics and health management community in terms of performance, ease of use, and flexibility of the explainable artificial intelligence method.
(iii) XAI as PHM tool and instrument of explanation: explainable artificial intelligence could be preferred or required within prognostics and health management compared to standalone methods.
(iv) PHM performance uninfluenced by XAI: The confidence of prognostics and health management practitioners and end users in the artificial intelligence model’s adoption should be boosted.
(v) Lack of human role, explanation metrics, and uncertainty management: Efforts need to be concentrated in these areas amongst other in the future. Moreover, the development of evaluation metrics that can cater prognostics and health management needs is urgently recommended.
(vi) Mostly real case studies were tested: the confidence of prognostics and health management practitioners and end users in the artificial intelligence model’s adoption should be increased.

The limitations of this study are stated below:

(i) This review does not classify explainable artificial intelligence methods in term of their nature (post-hoc, local, or global explainability): New insights or patterns could potentially be discovered by applying this classification.
(ii) The review does not explore in detail the subject of explainability metrics: This aspect should be a standalone subject as it is a vast and emerging topic that involves the explainable artificial intelligence methods, human factors, and the proper need for the domain.

## Data Availability

The data presented in this study are openly available online at: www.mdpi.com/ethics.github.com/Kamalnor?tab=repositories (accessed on 19 October 2021).

## Appendix A

**Table A1:** Analysis results of selected articles.

ID	Authors and Year	Title	Publisher, Publication Name	PHM Activity	XAI Approach	Performance	XAI Assist PHM	Metric	Human Role	Uncertainty Management	Case Study
1	[54] Wong et al., 2015	On equivalence of FIS and ELM for interpretable rule-based knowledge representation	IEEE, IEEE Transactions on Neural Networks and Learning Systems	Diagnostic	Rule- and knowledge-based	Accuracy: 85.14% Good	Yes	No	No	No	Real— Circulating cooling water system for turbine. (energy sector)
2	[55] Wu et al., 2018	K-PdM: KPI-oriented machinery deterioration estimation framework for oredictive maintenance using cluster-based hidden Markov model	IEEE, IEEE Access	Prognostic	Rule- and knowledge-based	RMSE: 14.28 Very Good	No	No	No	Probabilistic state transition model	Simulated—Turbofan engine (aerospace)
3	[56] Massimo et al., 2018	Unsupervised classification of multichannel profile data using PCA: An application to an emission control system	Elsevier, Computers and Industrial Engineering	Diagnostic	Cluster- based	MSE: 2.127 × 10^−5^ to 5.809 × 10^−3^ Very Good	Yes	No	Yes	No	Real—Emission control system (automotive, environment)
4	[57] Mathias et al, 2019	Forecasting remaining useful life: Interpretable deep learning approach via variational Bayesian inference	Elsevier, Decision Support Systems	Prognostic	Interpretable model	MAE: 13.267 Better than other methods, except LSTM	No	No	No	Uncertainty in model parameters	Simulated— Turbofan engine (Aerospace)
5	[58] Imene et al., 2019	Fault isolation in manufacturing systems based on learning algorithm and fuzzy rule selection	Springer, Neural Computing and Applications	Diagnostic	Rule- and knowledge- based	Accuracy: 97.01% Very Good	Yes	No	No	Probabilistic classification by Bayes decision rule	Real— Rotary kiln (civil engineering)
6	[59] Kerelous et al., 2019	Interpretable logic tree analysis: A data-driven fault tree methodology for causality analysis	Elsevier, Expert Systems with Applications	Diagnostic	LAD	Mean and standard errors are less than 2% and 1% Very good	Yes	No	Yes	FTA—Expert opinion	Simulated— Actuator system (manufacturing, energy, production, chemical)
7	[60] Rajendran et al., 2019	Unsupervised wireless spectrum anomaly detection with interpretable features	IEEE, IEEE Transactions on Cognitive Communications and Networking	Anomaly detection	Autoencoder	Generally better than other tested methods	Yes	No	No	Probabilistic classification error by discriminator	Real—software defined radio spectrum simulated—synthetic data (communication)
8	[61] Wang et al., 2019	An attention-augmented deep architecture for hard drive status monitoring in large-scale storag systems	ACM, ACM Transactions on Storage	Prognostic, diagnostic	Attention mechanism	Prognostic precision: 94.5–98.3% Generally, better than other methods. No comparison in diagnostic	Diag: Yes Prog: No	No	No	No	Real— Hard drive (information technology)
9	[62] Le et al., 2019	Visualization and explainable machine learning for efficient manufacturing and system operations	ASTM, Smart and Sustainable Manufacturing Systems	Diagnostic	Others	N/A ^1^	Yes	No	Yes	No	Simulated—turbofan (aerospace)
10	[63] Langone et al., 2020	Interpretable anomaly prediction: Predicting anomalous behavior in industry 4.0 settings via regularized logistic regression tools	Elsevier, Data and Knowledge Engineering	Anomaly detection	Interpretable model	Kappa: 0.4–0.6 AUC: 0.6–0.8 F1: 0.3–0.5 PRAUC: 0.2–0.4 Good	Yes	No	No	Statistical feature extraction	Real— High-pressure plunger pump (chemical)
11	[64] Peng et al., 2020	A dynamic structure-adaptive symbolic approach for slewing bearings life prediction under variable working conditions	Sage, Structural Health Monitoring	Prognostic	Interpretable model	RMSE: 18.19 Better than previous methods	Yes	No	No	No	Real— Slewing bearings (rotating machinery, energy, manufacturing)
12	[65] Ritto et al., 2020	Digital twin, physics-based model, and machine learning applied to damage detection in structures	Elsevier, Mechanical Systems and Signal Processing	Diagnostic	Interpretable model	Accuracy: 74.8–93.3% Good	No	No	No	No	Not specified— Spring mass system (wind turbine, energy)
13	[66] Rea et al., 2020	Progress toward interpretable machine learning based disruption predictors across tokamaks	Taylor and Francis, Fusion Science and Technology	Diagnostic	Interpretable model	N/A	No	No	No	Physic-based indicator	Real DIII—D and JET tokamaks (energy)
14	[67] Murari et al., 2020	Investigating the physics of tokamak global stability with interpretable ML tools	MDPI, Applied Sciences	Anomaly detection	Mathematic equation	Success Rate > 90% Very Good	No	No	No	No	Type unspecified—Tokamak (energy)
15	[68] Zhou et al., 2020	Fault diagnosis of gas turbine based on partly interpretable convolutional neural networks	Elsevier, Energy	Diagnostic	Tree-based	Accuracy: 95.52% Better than other tested methods	Yes	No	No	No	Simulated— Gas turbine model (energy)
16	[69] Zhou et al., 2020	Addressing noise and skewness in interpretable health-condition assessment by learning model confidence	MDPI, Sensors	Diagnostic	Rule- and knowledge- based	F1 Score: 0.8005 Very Good	No	No	No	No	Real— Aircraft structure. (aerospace)
17	[70] Jianbo et al., 2020	Knowledge extraction and insertion to deep belief network for gearbox fault diagnosis	Elsevier, Knowledge-Based Systems	Diagnostic	Rule- and Knowledge-based	Accuracy: 92.33 Very Good	Yes	No	No	No	Real— Gearbox (manufacturing, energy, automotive)
18	[71] Conde et al., 2020	Isotonic boosting classification rules	Springer, Advances in Data Analysis and Classification	Diagnostic	Rule- and knowledge-based	Total Misclassification Probability (TMP): 0.036-0.164 Good and comparable to other methods	Yes	No	No	No	Real— Induction motor (manufacturing, energy, production)
19	[72] Antonio et al., 2020	Using an autoencoder in the design of an anomaly detector for smart manufacturing	Elsevier, Pattern Recognition Letters	Anomaly detection	Autoencoder	Precision: 77.8–100% Accuracy: 94.9–100% Same as the previous best method	Yes	No	No	No	Simulated— Continuous batch washing equipment (industrial laundry)
20	[73] Abid et al., 2020	Robust interpretable deep learning for intelligent fault diagnosis of induction motors	IEEE, IEEE Transactions on Instrumentation and Measurement	Diagnostic	Filter-based	Accuracy: 99.95% ± 0.05% Better than other tested methods and previous works	Yes	No	No	No	Real— Electrical and mechanical motor (Manufacturing, Energy, Production)
21	[74] Liu et al., 2020	Tscatnet: An interpretable cross-domain intelligent diagnosis model with antinoise and few-shot learning capability	IEEE, IEEE Transactions on Instrumentation and Measurement	Diagnostic	Filter-based	Accuracy: 100% Better than other tested methods	Yes	No	No	No	Real—Bearing, drive train (manufacturing, energy, production)
22	[75] Li et al., 2020	Waveletkernelnet: an interpretable deep neural network for industrial intelligent diagnosis.	IEEE, IEEE Transactions on Systems, Man, and Cybernetics: Systems	Diagnostic	Filter-based	Accuracy: 92.61–99.91% Better than other tested methods	Yes	No	No	No	Real—Bearing, drive train (manufacturing, energy, production)
23	[76] Chen et al., 2020	Vibration signals analysis by explainable artificial intelligence approach: Application on bearing faults diagnosis	IEEE, IEEE Access	Diagnostic	Attention mechanism	N/A	No	No	No	No	Real— Rolling bearing (manufacturing, energy, production)
24	[77] Sun et al., 2020	Vision-based fault diagnostics using explainable deep learning with class activation maps	IEEE, IEEE Access	Diagnostic	Attention mechanism	Accuracy: 95.85% Precision: 100% Very good	No	No	No	No	Real— Base-excited cantilever beam, water pump system (manufacturing, energy, production)
25	[78] Oh et al., 2020	VODCA: Verification of diagnosis using CAM-based approach for explainable process monitoring	MDPI, Sensors	Diagnostic	Attention mechanism	Accuracy: 78.4–99.5% Good	Yes	No	No	True positive and true negative indicators	Simulated— Ford motor and real—sapphire grinding (automotive, production)
26	[79] Sreenath et al., 2020	Fouling modeling and prediction approach for heat exchangers using deep learning	Elsevier, International Journal of Heat and Mass Transfer	Failure Prediction	Model agnostic	Accuracy: 99.80–99.92% Very good	No	No	No	No	Simulated— Heat-exchanger model (manufacturing, energy, production)
27	[80] Hong et al., 2020	Remaining useful life prognosis for turbofan engine using explainable deep neural network with dimensional reduction	MDPI, Sensors	Prognostic	Model Agnostic	RMSE: 10.41 Very good	No	No	No	No	Simulated—Turbofan engine (aerospace)
28	[81] Grezmak et al., 2020	Interpretable convolutional neural network through layer-wise relevance propagation for machine fault diagnosis	IEEE, IEEE Sensors Journal	Diagnostic	LRP	Accuracy: 100% Very good	No	No	No	No	Real— Induction motor (manufacturing, energy, production)
29	[82] Ming et al., 2020	ProtoSteer: Steering deep sequence model with prototypes	IEEE, IEEE Transactions on Visualization and Computer Graphics	Diagnostic	Others	N/A	Yes	No	Yes	No	Real— Vehicle fault log (automotive)
30	[83] Chen et al., 2020	Frequency-temporal-logic-based bearing fault diagnosis and fault interpretation using Bayesian optimization &ANN	Elsevier, Mechanical Systems and Signal Processing	Diagnostic	Others	Better error percentage, error rate and robustness than other tested methods	Yes	No	No	No	Real—Bearings (manufacturing, energy, production)
31	[84] Steenwinckel et al., 2021	FLAGS: A methodology for adaptive anomaly detection and root cause analysis on sensor data streams by fusing expert knowledge with machine learning	Elsevier, Future Generation Computer Systems	Anomaly detection, diagnostic	Rule- and knowledge- based	Accuracy: 75% Good in anomaly detection, no result for diagnostic	Yes, for both	No	Yes	FMEA and FTA—Expert opinion	Real—Train (transportation)
32	[85] Zhang et al., 2021	A new interpretable learning method for fault diagnosis of rolling bearings	IEEE, EEE Transactions on Instrumentation and Measurement	Diagnostic	Cluster- based	Accuracy: 99.3–100% Very good	Yes	No	No	No	Real— Rolling bearing (manufacturing, energy, production)
33	[86] Onchis et al., 2021	Stable and explainable deep learning damage prediction for prismatic cantilever steel beam	Elsevier, Computers in Industry	Diagnostic	Model Agnostic	Accuracy for 19% damage: 75–92% Accuracy for 43% damage: 85–95% Good	Yes, by LIME only	Stability-fit compensation index (SFC)—Quality indicator of the explanations	No	Yes	Real— Prismatic cantilever steel beam (civil engineering, structural engineering)
34	[87] Kim et al., 2021	An explainable convolutional neural network for fault diagnosis in linear motion guide	IEEE, IEEE Transactions on Industrial Informatics	Diagnostic	Attention mechanism	Accuracy: 99.59–99.71% Very good	No	No	No	No	Real— Linear motion guide (manufacturing, energy, production)
35	[88] Ding et al., 2021	Stationary subspaces autoregressive with exogenous terms methodology for degradation trend estimation of rolling and slewing bearings	Elsevier, Mechanical Systems and Signal Processing	Prognostic	Others	MAE: 0.0375–0.0414 RMSE: 0.0482–0.0659 Better than other methods and comparable to previous works	Yes	No	No	No	Real— Rolling and slewing bearings (manufacturing, energy, production)

^1^ N/A = Item not included in the studied work.

**Table A2:** Excluded articles according to the publication year.

ID	Authors, Date	Title	Publisher, Publication Name	Exclusion Reason
1	[89] Kumar et al., 2016	Adaptive cluster tendency visualization and anomaly detection for streaming data	ACM, ACM Transactions on Knowledge Discovery from Data	Non-PHM-XAI implementation/case study
2	[90] Bao et al., 2016	Improved fault detection and diagnosis using sparse global-local preserving projections	Elsevier, Journal of Process Control	Process monitoring and anomaly detection
3	[91] Kozjek et al., 2017	Interpretative identification of the faulty conditions in a cyclic manufacturing process	Elsevier, Journal of Manufacturing Systems	Process monitoring and diagnosis
4	[92] Ragab et al., 2017	Fault diagnosis in industrial chemical processes using interpretable patterns based on logical analysis of data	Elsevier, Expert Systems with Applications	Process monitoring and fault diagnosis
5	[93] Tang et al., 2018	Fisher discriminative sparse representation based on DBN for fault diagnosis of complex system	MDPI, Applied Science	Process monitoring and fault diagnosis
6	[94] Luo et al., 2018	Knowledge-data-integrated sparse modeling for batch process monitoring	Elsevier, Chemical Engineering Science	Process anomaly detection and diagnosis
7	[95] Puggini et al., 2018	An enhanced variable selection and Isolation Forest based methodology for anomaly detection with OES data	Elsevier, Engineering Applications of Artificial Intelligence	Process anomaly detection and diagnosis
8	[96] Cheng et al., 2018	Monitoring influent measurements at water resource recovery facility using data-driven soft sensor approach	IEEE, IEEE Sensors Journal	Process anomaly detection
9	[97] Zhang et al., 2018	Weakly correlated profile monitoring based on sparse multi-channel functional principal component analysis	Taylor and Francis, IISE Transactions	Process monitoring
10	[98] Luo et al., 2018	Industrial process monitoring based on knowledge-data integrated sparse model and two-level deviation magnitude plots	ACS, Industrial and Engineering Chemistry Research	Process monitoring, anomaly detection and diagnosis
11	[99] Vojíř et al., 2018	EasyMiner.eu: web framework for interpretable machine learning based on rules and frequent item sets	Elsevier, Knowledge-Based Systems	Only development version offers anomaly detection
12	[100] Du et al., 2019	A condition change detection method for solar conversion efficiency in solar cell manufacturing processes	IEEE, IEEE Transactions on Semiconductor Manufacturing	Process monitoring and anomaly detection
13	[101] Keneniet et al., 2019	Evolving rule-based explainable artificial intelligence for unmanned aerial vehicles	IEEE, IEEE Access	Interpret why agent deviate from its mission, not because of system failure
14	[102] Wang et al., 2019	Dynamic soft sensor development based on convolutional neural networks	ACS, Industrial and Engineering Chemistry Research	Process modelling
15	[103] Wang et al., 2019	Explicit and interpretable nonlinear soft sensor models for influent surveillance at a full-scale wastewater treatment plant	Elsevier, Journal of Process Control	Process monitoring and variable prediction
16	[104] Liu et al., 2019	Intelligent online catastrophe assessment and preventive control via a stacked denoising autoencoder	Elsevier, Neurocomputing	Black-box
17	[105] Bukhsh et al., 2019	Predictive maintenance using tree-based classification techniques: a case of railway switches	Elsevier, Transportation Research Part C	Predict maintenance need, activity type and maintenance trigger status
18	[106] Ragab et al., 2019	Deep understanding in industrial processes by complementing human expertise with interpretable patterns of machine learning	Elsevier, Expert Systems with Applications	Process monitoring and fault diagnosis
19	[107] Luo et al., 2019	Sparse robust principal component analysis with applications to fault detection and diagnosis	ACS, Industrial and Engineering Chemistry Research	Process monitoring, fault detection and diagnosis
20	[108] Jie et al., 2020	Process abnormity identification by fuzzy logic rules and expert estimated thresholds derived certainty factor	Elsevier, Chemometrics and Intelligent Laboratory Systems	Process anomaly diagnosis
21	[109] Sajedi et al., 2020	Dual Bayesian inference for risk-informed vibration-based diagnosis	Wiley, Computer-Aided Civil and Infrastructure Engineering	Uncertainty interpretation, not model’s interpretation
22	[110] Sun et al., 2020	ALVEN: Algebraic learning via elastic net for static and dynamic nonlinear model identification	Elsevier, Computers and Chemical Engineering	Process monitoring and variable prediction
23	[111] Henriques et al., 2020	Combining k-means and XGBoost models for anomaly detection using log datasets	MDPI, Electronics	Anomaly in project, not engineered system
24	[112] Gorzałczany et al., 2020	A modern data-mining approach based on genetically optimized fuzzy systems for interpretable and accurate smart-grid stability prediction	MDPI, Energies	Electrical grid demand stability in financial perspective
25	[113] Müller et al., 2020	Data or interpretations impacts of information presentation strategies on diagnostic processes	Wiley, Human Factors and Ergonomics in Manufacturing and Service Industries	Experiment with operator effectivity following quality of interpretability
26	[114] Shriram et al., 2020	Least squares sparse principal component analysis and parallel coordinates for real-time process monitoring	ACS, Industrial and Engineering Chemistry Research	Process monitoring and diagnosis
27	[115] Alshraideh et al., 2020	Process control via random forest classification of profile signals: an application to a tapping process	Elsevier, Journal of Manufacturing Processes	Process monitoring and anomaly detection
28	[116] Minghua et al., 2020	Diagnosing root causes of intermittent slow queries in cloud databases	ACM, Proceedings of the VLDB Endowment	Diagnosing slow query due to lack of resources, not failure
29	[117] Shaha et al., 2020	Performance prediction and interpretation of a refuse plastic fuel fired boiler	IEEE, IEEE Access	Performance prediction
30	[118] Kovalev et al., 2020	SurvLIME: a method for explaining machine learning survival models	Elsevier, Knowledge-Based Systems	Medical survival model
31	[119] Kovalev et al., 2020	A robust algorithm for explaining unreliable machine learning survival models using the Kolmogorov.Smirnov bounds	Elsevier, Neural Networks	Medical survival model
32	[120] Karn et al., 2021	Cryptomining detection in container clouds using system calls and explainable machine learning	IEEE, IEEE Transactions on Parallel and Distributed Systems	Network attack
33	[121] Gyula et al., 2021	Decision trees for informative process alarm definition and alarm-based fault classification	Elsevier, Process Safety and Environmental Protection	Process monitoring and anomaly detection
34	[122] Zaman et al., 2021	Fuzzy heuristics and decision tree for classification of statistical feature-based control chart patterns	MDPI, Symmetry	Process monitoring and diagnosis
35	[123] Li et al., 2021	DTDR-ALSTM: Extracting dynamic time-delays to reconstruct multivariate data for improving attention-based LSTM industrial time series prediction models	Elsevier, Knowledge-Based Systems	Process monitoring and variable prediction

**Table A3:** Search strategy.

Database and Date	Number of Extracted Papers	Search Field and Keywords	Filters Applied
IEEE Xplore 18/02/21	144	** Using ‘Document Title’**: 1. **Document Title**: explainable OR **Document Title**: interpretable, **Search within results**: diagnostic 2. **Document Title**: explainable OR **Document Title**: interpretable, **Search within results**: prognostic 3. **Document Title**: explainable OR **Document Title**: interpretable, **Search within results**: diagnosis 4. **Document Title**: explainable OR **Document Title**: interpretable, **Search within results**: prognosis 5. **Document Title**: explainable OR **Document Title**: interpretable, **Search within results**: anomaly detection 6. **Document Title**: explainable OR **Document Title**: interpretable, **Search within results**: RUL 7. **Document Title**: explainable OR **Document Title**: interpretable, **Search within results**: remaining useful life 8. **Document Title**: explainable AI OR **Document Title**: explainable machine learning OR **Document Title**: explainable deep learning OR **Document Title**: XAI, **Search within results**: prognostic 9. **Document Title**: explainable AI OR **Document Title**: explainable machine learning OR **Document Title**: explainable deep learning OR **Document Title**: XAI, **Search within results**: diagnostic 10. **Document Title**: explainable AI OR **Document Title**: explainable machine learning OR **Document Title**: explainable deep learning OR **Document Title**: XAI, **Search within results**: diagnosis 11. **Document Title**: explainable AI OR **Document Title**: explainable machine learning OR **Document Title**: explainable deep learning OR **Document Title**: XAI, **Search within results**: prognosis 12. **Document Title**: explainable AI OR **Document Title**: explainable machine learning OR **Document Title**: explainable deep learning OR **Document Title**: XAI, **Search within results**: anomaly detection 13. **Document Title**: explainable AI OR **Document Title**: explainable machine learning OR **Document Title**: explainable deep learning OR **Document Title**: XAI, **Search within results**: RUL 14. **Document Title**: explainable AI OR **Document Title**: explainable machine learning OR **Document Title**: explainable deep learning OR **Document Title**: XAI, **Search within results**: remaining useful life 15. **Document Title**: interpretable AI OR **Document Title**: interpretable machine learning OR **Document Title**: interpretable deep learning OR **Document Title**: XAI, **Search within results**: diagnostic 16. **Document Title**: interpretable AI OR **Document Title**: interpretable machine learning OR **Document Title**: interpretable deep learning OR **Document Title**: XAI, **Search within results**: prognostic 17. **Document Title**: interpretable AI OR **Document Title**: interpretable machine learning) OR **Document Title**: interpretable deep learning OR **Document Title**: XAI, **Search within results**: prognosis 18. **Document Title**: interpretable AI OR **Document Title**: interpretable machine learning) OR **Document Title**: interpretable deep learning OR **Document Title**: XAI, **Search within results**: diagnosis 19. **Document Title**: interpretable AI OR **Document Title**: interpretable machine learning) OR **Document Title**: interpretable deep learning OR **Document Title**: XAI, **Search within results**: anomaly detection 20. **Document Title**: interpretable AI OR **Document Title**: interpretable machine learning) OR **Document Title**: interpretable deep learning OR **Document Title**: XAI, **Search within results**: RUL 21. **Document Title**: interpretable AI OR **Document Title**: interpretable machine learning) OR **Document Title**: interpretable deep learning OR **Document Title**: XAI, **Search within results**: remaining useful life **Using ‘Abstract’**: 22. **Abstract**: explainable AI OR **Abstract**: explainable machine learning OR **Abstract**: explainable deep learning OR **Abstract**: XAI, **Search within results**: prognostic 23. **Abstract**: explainable AI OR **Abstract**: explainable machine learning OR **Abstract**: explainable deep learning OR **Abstract**: XAI, **Search within results**: diagnostic 24. **Abstract**: explainable AI OR **Abstract**: explainable machine learning OR **Abstract**: explainable deep learning OR **Abstract**: XAI, **Search within results**: diagnosis 25. **Abstract**: explainable AI OR **Abstract**: explainable machine learning OR **Abstract**: explainable deep learning OR **Abstract**: XAI, **Search within results**: prognosis 26. **Abstract**: explainable AI OR **Abstract**: explainable machine learning OR **Abstract**: explainable deep learning OR **Abstract**: XAI, **Search within results**: anomaly detection 27. **Abstract**: explainable AI OR **Abstract**: explainable machine learning OR **Abstract**: explainable deep learning OR **Abstract**: XAI, **Search within results**: RUL 28. **Abstract**: explainable AI OR **Abstract**: explainable machine learning OR **Abstract**: explainable deep learning OR **Abstract**: XAI, **Search within results**: remaining useful life 29. **Abstract**: interpretable AI OR **Abstract**: interpretable machine learning) OR **Abstract**: interpretable deep learning OR **Abstract**: XAI, **Search within results**: prognostic 30. **Abstract**: interpretable AI OR **Abstract**: interpretable machine learning) OR **Abstract**: interpretable deep learning OR **Abstract**: XAI, **Search within results**: diagnostic 31. **Abstract**: interpretable AI OR **Abstract**: interpretable machine learning) OR **Abstract**: interpretable deep learning OR **Abstract**: XAI, **Search within results**: prognosis 32. **Abstract**: interpretable AI OR **Abstract**: interpretable machine learning) OR **Abstract**: interpretable deep learning OR **Abstract**: XAI, **Search within results**: diagnosis 33. **Abstract**: interpretable AI OR **Abstract**: interpretable machine learning) OR **Abstract**: interpretable deep learning OR **Abstract**: XAI, **Search within results**: anomaly detection 34. **Abstract**: interpretable AI OR **Abstract**: interpretable machine learning) OR **Abstract**: interpretable deep learning OR **Abstract**: XAI, **Search within results**: RUL 35. **Abstract**: interpretable AI OR **Abstract**: interpretable machine learning) OR **Abstract**: interpretable deep learning OR **Abstract**: XAI, **Search within results**: remaining useful life	Journals, Early Access Article, **Specify Year Range**: 2015–2021
Science Direct 17/02/21	607	** Using ‘Title, abstract or author-specified keywords’**: 36. (“explainable” OR “interpretable”) AND (“prognostic” OR “diagnostic” OR “prognosis” OR “diagnosis” OR “anomaly detection” OR “RUL” OR “remaining useful life”) 37. (“explainable AI” OR “explainable machine learning” OR “explainable deep learning” OR “XAI”) AND (“prognostic” OR “diagnostic” OR “anomaly detection” OR “RUL” OR “remaining useful life”) 38. (“explainable AI” OR “explainable machine learning” OR “explainable deep learning” OR “XAI”) AND (“prognosis” OR “diagnosis” OR “anomaly detection” OR “RUL” OR “remaining useful life”) 39. (“interpretable AI” OR “interpretable machine learning” OR “interpretable deep learning” OR “XAI”) AND (“prognostic” OR “diagnostic” OR “anomaly detection” OR “RUL” OR “remaining useful life”) 40. (“interpretable AI” OR “interpretable machine learning” OR “interpretable deep learning” OR “XAI”) AND (“prognosis” OR “diagnosis” OR “anomaly detection” OR “RUL” OR “remaining useful life”)	**Article type**: Research Articles, **Subject areas**: Engineering and Computer Science, **Years**: 2015–2021
Springer Link 22/02/21	291	** Using ‘With all the words’**: 41. “explainable” OR “interpretable” AND “prognos” 42. “explainable” OR “interpretable” AND “prognos” 43. “explainable” OR “interpretable” AND “diagnos” 44. “explainable” OR “interpretable” AND “diagnos” 45. “explainable” OR “interpretable” AND “RUL” 46. “explainable” OR “interpretable” AND “RUL” 47. “explainable” OR “interpretable” AND “remaining useful life” 48. “explainable” OR “interpretable” AND “remaining useful life” 49. “explainable” OR “interpretable” AND “anomaly detection” 50. “explainable” OR “interpretable” AND “anomaly detection”	**Content Type**: Article, **Discipline**: Computer Science or Engineering, **Language**: English, **Show documents published**: 2015–2021
ACM Digital Library 28/05/21	75	** Using ‘Publication Title, Abstract and Keywords’**: 51. **Publication Title**: explainable or interpretable AND **Publication Title**: (prognos OR diagnos OR “anomaly detection” OR RUL OR “remaining useful life” 52. **Abstract**: explainable or interpretable AND **Abstract**: (prognos OR diagnos OR “anomaly detection” OR RUL OR “remaining useful life” 53. **Keywords**: explainable or interpretable AND **Keywords**: (prognos OR diagnos OR “anomaly detection” OR RUL OR “remaining useful life”	**Publications**: Journal, **Content Type**: Research Article, **Publication Date**: 2015–2021
Scopus 27/02/21	1931	54. (“explainable” OR “interpretable”) AND (“prognostic” OR “diagnostic” OR “prognosis” OR “diagnosis” OR “anomaly detection” OR “RUL” OR “remaining useful life”)	**Limited to**: Article, **Publication stage**: Final, **Subject Area**: Engineering and Comput Science, **Language**: English, **Exclude**: Medical, **Published from**: 2015–2021

**Table A4:** Value and classification of the indicated metric.

	Value	<50%	50–75%	75–90%	90–100%
Metric					
Accuracy		Bad	Fair	Good	Very good
Precision		Bad	Fair	Good	Very good
	**Value**	**0.00**–**0.20**	**0.21**–**0.40**	**0.41**–**0.6**	**0.61**–**1.00**
**Metric**					
F1		Bad	Fair	Good	Very good
AUC		Bad	Fair	Good	Very good
PRAUC		Bad	Fair	Good	Very good
Kappa		Bad	Fair	Good	Very good
